# Child diet and mother–child interactions mediate intervention effects on child growth and development

**DOI:** 10.1111/mcn.13308

**Published:** 2021-12-14

**Authors:** Lilia Bliznashka, Dana C. McCoy, Saima Siyal, Christopher R. Sudfeld, Wafaie W. Fawzi, Aisha K. Yousafzai

**Affiliations:** ^1^ Department of Global Health and Population Harvard T.H. Chan School of Public Health Boston Massachusetts USA; ^2^ Harvard Graduate School of Education Harvard University Cambridge Massachusetts USA; ^3^ Harvard Graduate School of Education Aga Khan University Karachi Pakistan; ^4^ Department of Nutrition Harvard T.H. Chan School of Public Health Boston Massachusetts USA; ^5^ Department of Epidemiology Harvard T.H. Chan School of Public Health Boston Massachusetts USA

**Keywords:** child, child development, developing countries, diet, mediation analysis, mother–child interactions, Pakistan

## Abstract

This study examined whether child diet and mother–child interactions mediated the effects of a responsive stimulation and nutrition intervention delivered from 2009 to 2012 to 1324 children aged 0–24 months living in rural Pakistan. Results showed that the intervention improved children's cognitive, language and motor development through child diet and mother–child interactions. Although the intervention did not improve child growth or socio‐emotional development, we observed positive indirect effects on child growth via child diet and on socio‐emotional development via both child diet and mother–child interactions. In addition, child diet emerged as a shared mechanism to improve both child growth and development, whereas mother–child interactions emerged as a distinct mechanism to improve child development. Nevertheless, our results suggest the two mechanisms were mutually reinforcing and that interventions leveraging both mechanisms are likely to be more effective at improving child outcomes than interventions leveraging only one of these mechanisms.

## Key messages

- A responsive stimulation and nutrition intervention delivered to young children from birth to 24 months of age in rural Pakistan improved child cognitive, language, and motor development at 24 months, but not child growth or socio‐emotional development.
- Child diet and mother–child interactions mediated intervention effects on child cognitive, language and motor development.
- Although we found no total intervention effects on child growth and socio‐emotional development, we observed positive indirect effects on socio‐emotional development through both child diet and mother–child interactions, and positive indirect effects on child growth through child diet.
- Leveraging both child diet and mother–child interactions as mechanisms may help enhance intervention effects on child outcomes.

## Graphical abstract

The contents of this page will be used as part of the graphical abstract of html only. It will not be published as part of main article.

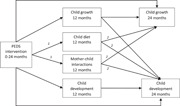

A responsive stimulation and nutrition intervention delivered to children 0–24 months of age in rural Pakistan improved children's cognitive, language and motor development throug child diet and mother‐child interactions. Although the intervention did not improve child growth or socio‐emotional development, we observed positive indirect effects on child growth via child diet and on socio‐emotional development via both child diet and mother‐child interactions. Interventions leveraging both mechanisms are likely to be more effective at improving child outcomes than interventions leveraging only one of these mechanisms.

## 1 INTRODUCTION

In low‐ and middle‐income countries (LMICs), 40% of preschool‐aged children are at risk of poor child development (Lu et al., 2020) and 22% are stunted (UNICEF et al., 2021). Community‐based interventions integrating responsive care, stimulation, nutrition and health components delivered to parents are recognized as effective strategies to improve child growth and development in early life (Jeong et al., 2021; Prado, Larson, et al., 2019; World Health Organization, 2020). Evidence suggests that interventions providing multiple inputs are needed to improve multiple child outcomes rather than interventions providing individual inputs or the standard of care (World Health Organization, 2020). Although evidence on what interventions work to improve child growth and development is expanding, less is known about how these interventions operate, the specific aspects of nurturing care these interventions benefit, and whether these benefits translate into improved child outcomes (Obradović et al., 2016; Yousafzai et al., 2014).

Prior studies in LMICs have examined intervention mechanisms such as maternal mental health (Aboud et al., 2013; Brown et al., 2017; Chang et al., 2015; Hamadani et al., 2019; Yousafzai et al., 2015), parental stimulation (Aboud et al., 2013; Chang et al., 2015; S. Grantham‐McGregor et al., 2020; Hamadani et al., 2019; Jeong et al., 2019; Obradović et al., 2016; Yousafzai et al., 2015) and maternal parenting knowledge (Aboud & Yousafzai, 2015; Aboud et al., 2013; Attanasio et al., 2018; Chang et al., 2015; S. Grantham‐McGregor et al., 2020; Hamadani et al., 2019). However, other pathways remain empirically understudied. A small number of studies have assessed mother–child interactions (Aboud & Akhter, 2011; Brown et al., 2017; Murray et al., 2016; Obradović et al., 2016; Yousafzai et al., 2015) and child diet (Aboud et al., 2013; Frongillo et al., 2017; Luoto et al., 2021; Yousafzai et al., 2015) as potential means through which multi‐input interventions might influence child outcomes. Although these studies build our emerging understanding on the importance of these mechanisms, most were not explicitly designed to study indirect effects or multi‐input approaches. Specifically, most prior studies have either assessed direct intervention effects on these mechanisms without formally conducting mediation analysis (thus not quantifying indirect effects), considered only single input approaches (i.e., stimulation or nutrition alone, but not integrated), or have only examined one or two child outcomes. Therefore, child diet and mother–child interactions remain to be unpacked as mechanisms through which multi‐input interventions in LMICs improve multiple facets of child growth and development.

Child diet can affect cognitive development both directly through brain development during infancy, and indirectly by affecting child health, physical activity, and caregiver behaviour (Prado & Dewey, 2014). Although nutrition interventions can improve both linear growth and, to a lesser extent, child development (Prado, Larson, et al., 2019), it is unclear whether these impacts are achieved primarily through child diet or whether other mechanisms, such as caregiver behaviour, are also at work. Limited evidence from Bangladesh suggests that child diet mediates the effects of nutrition interventions on child growth and development (Frongillo et al., 2017; Vazir et al., 2013). Further, a recent study in Kenya showed that child diet together with parental stimulation, maternal knowledge and recall of intervention messages mediated the effects of a responsive stimulation and nutrition intervention on child cognitive and receptive language development (Luoto et al., 2021). However, to our knowledge, no studies have assessed whether responsive stimulation interventions in LMICs work to improve child growth and development exclusively through child diet.

Maternal responsive behaviours and developmentally appropriate and supportive mother–child interactions are important for child health, nutrition, and development in early life (Eshel et al., 2006). Responsive interactions involve a three‐step process: observation of the child's signals, interpretation of these signals, and action/response to meet the child's signals appropriate for the developmental age of the child (Eshel et al., 2006). A caregiver may also demonstrate a range of developmentally supportive interactions, that are not necessarily responsive, but nurture development (e.g., directive interactions, language inputs). Given the complex nature of maternal responsive behaviours, culturally relevant measures with tested reliability and validity for use in LMICs are limited and no gold standard of measuring responsive behaviours exists (Bentley et al., 2011; Hentschel et al., 2021; Pérez‐Escamilla & Segura‐Pérez, 2020). As the ‘interpretation’ step of the process is difficult to observe, most measures focus on the child's signals and maternal responses (Hentschel et al., 2021). The few studies in LMICs that have collected data on caregiver‐child interactions tend to encompass both maternal responsive behaviours and supportive mother–child interactions, which are often assessed in a picture book‐reading context (Aboud & Akhter, 2011; Betancourt et al., 2020; Brown et al., 2017; Knauer et al., 2020; Murray et al., 2016; Obradović et al., 2016; Scherer et al., 2019). Evidence from these studies suggests that improvements in mother–child interactions mediate the positive effects of parenting and responsive stimulation interventions on child growth and development (Aboud & Akhter, 2011; Brown et al., 2017; Eshel et al., 2006; Landry et al., 2006; Murray et al., 2016; Obradović et al., 2016). In addition, limited evidence indicates that mother–child interactions mediate the effects of nutrition interventions in early life on child growth at 4 years of age (Brown et al., 2017). Whether mother–child interactions mediate the effect of responsive stimulation and nutrition interventions in LMICs has not been empirically tested in children less than 2 years of age.

Understanding how multi‐input interventions achieve impact on child outcomes can help increase intervention effectiveness by leveraging common and mutually reinforcing mechanisms. Therefore, in the present study we sought to deepen knowledge regarding the mechanisms through which multi‐input interventions in LMICs may affect child outcomes in early life. Specifically, we used data from the Pakistan Early Child Development Scale‐up (PEDS) intervention trial to explore child diet and mother–child interactions at 1 year of age as potential mediators that might explain the effects of a responsive stimulation and nutrition intervention on child growth and development at 2 years of age.

## 2 METHODS

### 2.1 PEDS trial

The PEDS intervention trial was designed to improve child growth and development by improving the nurturing care environment in which children thrive. PEDS was a longitudinal cluster‐randomized trial evaluating the effectiveness of integrating responsive stimulation (RS) and enhanced nutrition (EN) interventions into The National Program for Family Planning and Primary Healthcare [known as the Lady Health Worker (LHW) program] conducted in Sindh, Pakistan (2009–2012). The interventions and study design have been described in detail elsewhere (Yousafzai et al., 2014). Briefly, 80 clusters (the catchment area of LHWs) were randomized into one of four intervention arms (20 clusters per arm): (1) RS, (2) EN, (3) RS + EN, and (4) Control. The standard of care received by the control arm involved basic nutrition, health, and hygiene education. In addition to the standard of care, the RS arms received a locally adapted version of the UNICEF and WHO Care for Child Development package, which promotes sensitive and responsive parenting through developmentally appropriate play and talk activities (i.e., responsive stimulation). The EN arms received enhanced nutrition education, which built on the standard of care by including information on the association between good nutrition and health, counselling on responsive feeding, and problem solving about feeding. Children in the EN arms also received multiple micronutrient supplementation from 6 to 24 months of age. In each intervention arm, LHWs conducted monthly home visits to deliver the intervention. The RS intervention arms also received monthly community group sessions to support the delivery of the RS package. Mother‐child pairs were enroled at birth (child was <2.5 months) and received the intervention until the child reached 24 months of age. The co‐primary outcomes of the trial were child development and linear growth at 24 months.

Prior work has shown that the RS and RS + EN interventions improved children's cognitive, language, and motor development at 24 months, whereas the EN intervention improved language development at 24 months and child linear growth from birth to 24 months (Yousafzai et al., 2014). The PEDS cohort was followed‐up when children were 4 years old, 2 years after the intervention ended. Using data from the PEDS trial and this follow‐up, N. Brown et al. (2017) showed that improved maternal mental health and mother–child interactions mediated RS and EN effects on child growth at 4 years of age. Obradović et al. (2016) showed that the RS intervention also maintained its positive effects on child cognitive development and executive function at 4 years of age through improved parental stimulation (i.e., parental engagement in play to support children's development) and scaffolding behaviours (i.e., parental support and guidance to help a child learn new age‐appropriate skills). Lastly, Jeong et al. (2019) also showed that parental stimulation mediated RS effects on cognitive and socio‐emotional development at 4 years of age.

We build directly onto this prior PEDS work by examining mother–child interactions as a mechanism to improve child growth and development in the first 2 years of life rather than later in life. In addition, we assessed the RS and EN interventions alone and in combination, which has not been previously done. Expanding prior work, we also examined whether the interventions worked through child diet to improve child growth and development outcomes.

### 2.2 Conceptual framework

**Figure 1:**
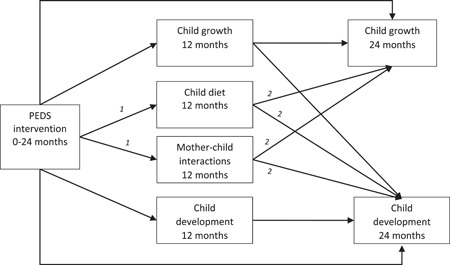
Conceptual framework showing the Pakistan Early Child Development Scale‐up Trial (PEDS) intervention effects on child growth and development through child diet and mother–child interactions. The numbers describe the direct effects comprising the indirect effects of interest we assessed: (1) direct intervention effects on mediators at 12 months of age, (2) prospective associations between the mediators at 12 months of age and child outcomes at 24 months of age

Figure 1 presents our conceptual framework, based on prior models (Brown & Pollitt, 1996; Prado et al., 2017). Positive direct intervention effects on child diet and mother–child interactions at 12 months (see #1 paths in Figure 1) were previously established (Yousafzai et al., 2015). We hypothesized positive prospective associations between child diet and mother–child interactions at 12 months and child growth and development at 24 months (see #2 paths in Figure 1), a hypothesis supported by several studies in LMICs (Eshel et al., 2006; Iannotti et al., 2016; Pollitt et al., 2000; Prado, Yakes Jimenez, et al., 2019). Based on the evidence presented above, we also hypothesized positive indirect intervention effects on child growth and development at 24 months through child diet and mother–child interactions. As significant indirect effects are possible in the context of overall null effects [due to competitive mediation, type 1 or type 2 error, or differential power to detect these effects (Fairchild & McDaniel, 2017; Rucker et al., 2011; Zhao et al., 2010)], we examined all co‐primary outcomes regardless of whether the intervention impacted them or not.

### 2.3 Participants

The analytic sample included 1350 mother–child pairs assessed at enrolment, 12 and 24 months, which represented 91% of the enrolment sample. Participants were excluded due to child death (4.4%), migration outside of the study area (4.3%), or refusal (0.8%). Children included in the analytic sample and those excluded were generally similar in terms of enrolment characteristics (Table S1). Therefore, the risk of bias in our sample appeared to be minimal.

Trained enumerators interviewed the child's mother during home visits and measured child length and weight. Trained community‐based child development assessors (CCDAs) conducted the child development and mother–child interactions assessments during home visits. Enumerators and CCDAs were masked to the intervention and were rotated to reduce familiarity with the families and villages. Further details on the training of enumerators and CCDAs are available in Yousafzai et al. (2014).

### 2.4 Measures

Intervention exposure was based on the intention‐to‐treat principle. We included three binary variables for each intervention arm: RS, EN, RS + EN. Each intervention arm was compared with the control arm.

Child growth was assessed at enrolment, 12 and 24 months using length‐for‐age *z*‐scores (LAZ), calculated according to the WHO Child Growth Standards (World Health Organization, 2006). Child development was assessed at 12 and 24 months of child age using the cognitive, language, motor, and socio‐emotional scales of the Bayley Scales of Infant and Toddler Development, Third Edition (BSID‐III) (Bayley, 2006). We calculated mean composite scores (*M* = 100, SD = 15) based on a conversion from raw scores to scaled composite scores. For a discussion on cultural adaptation and reliability see Yousafzai et al. (2014).

Child diet was assessed at 12 months using the WHO dietary diversity score (DDS) indicators (World Health Organization et al., 2010). Mothers reported on the foods the child consumed in the past 24 h. We created binary indicators for whether the child consumed each of seven food groups (grains/roots/tubers, legumes and nuts, dairy, eggs, flesh foods, vitamin A‐rich fruits and vegetables, and other fruits and vegetables) and summed them to create DDS (range 0–7).

Mother–child interactions at 12 months was assessed using the Observation of Mother‐Child Interaction (OMCI) tool (Rasheed & Yousafzai, 2015). The OMCI was designed to capture maternal responsive behaviours and developmentally supportive interactions, including contingent responding, emotional‐affective support, support for infant foci of attention, and language inputs. It consists of 19 items: 12 items for the mother, 6 items for the child, and 1 item on mutual enjoyment. As the reciprocity of the mother–child interactions is key (Black & Aboud, 2011), we used all 19 items in the analysis. The CCDAs observed a live 5‐min interaction between the mother and child using a picture book, and rated the frequency of the 19 behaviours using the following scoring criteria: 0 = *never occurred*, 1 = *occurred infrequently (1–2 times)*, 2 = *occurred sometimes (3–4 times)*, 3 = *occurred frequently (5+ times)*. Total OMCI score (theoretical range 0–57) was calculated by summing the scores on the 19 items. Higher OMCI scores indicate higher frequency of positive interactions and behaviours. Descriptive statistics for the 19 items are shown in Table S2. Further details on the OMCI are available in Rasheed and Yousafzai (2015).

To minimize potential confounding, household wealth and food security, maternal education and mental health, and child age, sex and number of siblings (a proxy for household size) at enrolment were included as covariates. Household wealth was entered as a factor score calculated using principal components analysis based on 44 items assessing property and livestock ownership, and water and electricity access. Household food security was defined based on the Household Food Insecurity Access Scale (Coates et al., 2007). Maternal mental health was assessed using the self‐reported questionnaire (SRQ‐20) (World Health Organization, 1994).

Biserial correlations for the analysed variables are shown in Table S3. We observed significant correlations between OMCI and DDS at 12 months and all outcomes at 24 months, lending support to our hypotheses of prospective relations between the mediators and the outcomes.

### 2.5 Statistical analysis

We used a longitudinal cross‐lagged structural equation model (Kline, 2015) to examine the total, direct and indirect effects of the PEDS intervention on child growth and development via child diet and mother–child interactions outlined in Figure 1. Assuming sequential ignorability, the indirect effects we estimate are equivalent to average causal mediation effects (Emsley et al., 2010; Imai et al., 2010; VanderWeele, 2012). The model was fit separately for each BSID‐III scale as prior evidence indicated that RS and EN inputs were differentially associated with each domain. Standard errors were clustered at the LHW level. Bias‐corrected (BC) bootstrapping with 5000 draws was used to test the significance of the total, direct and indirect effects (Preacher & Hayes, 2008). We present BC bootstrapped 95% confidence intervals (CI) throughout referred to as 95% CI for brevity. Missing data on endogenous variables (child growth and development at 12 and 24 months, and child diet and mother–child interactions at 12 months) was handled by use of a full weight matrix. Observations with missing data on exogenous variables (*N* = 26) were excluded, reducing the effective sample size to 1324 children. Models were estimated in MPlus Version 8. Absolute model fit was determined acceptable based on the following criteria: comparative fit index (CFI) ≥ 0.90, root mean square error of approximation (RMSEA) ≤ 0.08, and standardized root mean squared residual (SRMR) ≤ 0.08 (Hu & Bentler, 1999).

### 2.6 Ethical considerations

Ethical approval was obtained from the institutional review board of the Aga Khan University in Pakistan. The technical steering committee of The National Program for Family Planning and Primary Healthcare (also known as the Lady Health Worker program) also provided permission for the trial. Written informed consent was obtained from all participants.

## 3 RESULTS

Table 1 shows child and mother characteristics at enrolment, 12 and 24 months. At enrolment, 25% of children were stunted (LAZ <−2 SD) and this proportion increased to 62% at 24 months. Child development scores were below the reference mean of 100 for all sub‐scales at both 12 and 24 months. At 12 months, 83% of children were still breastfed. Child diet was poor with children consuming primarily grains, roots, and tubers. Only 23% of children met a minimally diverse diet (DDS ≥ 4) at 12 months.

**Table 1:** Sample characteristics

Variables	*N*	% or mean ± SD
*Child, maternal, and household characteristics at enrolment*		
Child age (in months)	1350	0.68 ± 0.65
Child is a boy	1350	54
Mother has no formal education	1350	68
Mother's SRQ‐20 (range 0–20)	1350	6.57 ± 3.89
Household wealth index	1342	0.01 ± 1.00
Household is food secure	1348	68
Number of siblings	1341	2.51 ± 2.30
*Intervention group assignment*		
Responsive stimulation (RS)	1350	26
Enhanced nutrition (EN)	1350	24
RS + EN	1350	25
Control	1350	25
*Mediators at 12 months*		
Child is currently breastfed	1338	83
Child consumed grains, roots, and tubers in past 24 h	1343	96
Child consumed legumes and nuts in past 24 h	1343	16
Child consumed flesh foods in past 24 h	1343	26
Child consumed eggs in past 24 h	1343	09
Child consumed dairy in past 24 h	1343	19
Child consumed vitamin A‐rich fruits and vegetables in past 24 h	1343	52
Child consumed other fruits and vegetables in past 24 h	1343	46
Child dietary diversity score (DDS, range 0‐7)	1343	2.64 ± 1.29
Child met minimum dietary diversity (DDS ≥ 4)	1343	23
Observation of mother–child interactions score (range 0–57)	1339	29.79 ± 8.63
*Outcomes*		
Length‐for‐age *z*‐score at enrolment	1340	−1.13 ± 1.41
Length‐for‐age *z*‐score at 12 months	1330	−1.99 ± 1.23
Length‐for‐age *z*‐score at 24 months	1321	−2.34 ± 1.12
Stunting (length‐for‐age *z*‐score < −2 SD) at enrolment	1340	25
Stunting (length‐for‐age *z*‐score < −2 SD) at 12 months	1330	48
Stunting (length‐for‐age *z*‐score < −2 SD) at 24 months	1321	62
Cognitive development at 12 months	1340	94.62 ± 13.81
Cognitive development at 24 months	1346	78.07 ± 14.65
Language development at 12 months	1340	74.88 ± 13.97
Language development at 24 months	1346	82.59 ± 13.59
Motor development at 12 months	1340	82.28 ± 13.68
Motor development at 24 months	1346	88.49 ± 17.32
Socio‐emotional development at 12 months	1340	79.08 ± 13.70
Socio‐emotional development at 24 months	1346	93.44 ± 18.33

Our model showed adequate fit for all four domains, with the exception of RMSEA in the motor and socio‐emotional development models (Table 2). Nevertheless, RMSEA was still close to the pre‐specified cut‐off for acceptable model fit.

**Table 2:** Model fit statistics

Model	*χ* ^2^ (df)	Comparative fit index	Root mean square error of approximation	Standardized root mean squared
Cognitive development	66.661*** (10)	0.968	0.065	0.026
Language development	44.595*** (10)	0.982	0.051	0.022
Motor development	120.515*** (10)	0.943	0.091	0.031
Socio‐emotional development	102.826*** (10)	0.943	0.084	0.030

*** *p* < 0.001.

All interventions improved cognitive, language, and motor scores at 24 months (Table 3) and DDS and OMCI at 12 months (Table S4). None of the interventions improved socio‐emotional scores or LAZ at 24 months (Table 3). All of these results were consistent with prior findings from the trial (Yousafzai et al., 2014, 2015). Higher OMCI at 12 months predicted higher development scores in all four domains at 24 months, whereas higher DDS at 12 months predicted higher LAZ, cognitive and language scores at 24 months (Table S4).

**Table 3:** Standardized total intervention effects on child growth and development at 24 months

		Bias‐corrected bootstrapped 95% CI	
Paths	*β*	Lower limit	Upper limit
*Total RS effects*			
RS → LAZ	−0.044	−0.122	0.032
RS → Cognitive development	0.328	0.246	0.410
RS → Language development	0.321	0.239	0.405
RS → Motor development	0.263	0.186	0.344
RS → Socio‐emotional development	0.000	−0.072	0.070
*Total EN effects*			
EN → LAZ	0.016	−0.083	0.106
EN → Cognitive development	0.111	0.016	0.197
EN → Language development	0.211	0.120	0.307
EN → Motor development	0.119	0.038	0.198
EN → Socio‐emotional development	0.074	0.000	0.152
*Total RS + EN effects*			
RS + EN → LAZ	−0.029	−0.101	0.043
RS + EN → Cognitive development	0.229	0.147	0.303
RS + EN → Language development	0.285	0.193	0.374
RS + EN → Motor development	0.119	0.124	0.271
RS + EN → Socio‐emotional development	0.030	−0.049	0.108

*Note*: The null hypothesis was *β* = 0. Models for each child development domain were fit separately. Models controlled for the following enrolment characteristics: household wealth, household food security, maternal education, maternal mental health, child age, child sex, child length‐for‐age *z*‐score and number of siblings. Models accounted for clustering and missing values.

Abbreviations: EN, enhanced nutrition; LAZ, length‐for‐age *z*‐score; RS, responsive stimulation.

After controlling for child diet and mother–child interactions as mediators, the RS and RS + EN interventions directly predicted lower LAZ at 24 months (Table S5). There was some indication that the RS intervention predicted lower socio‐emotional scores; however, the confidence interval was just above the null. In addition, the RS and RS + EN interventions improved cognitive, language and motor scores at 24 months, whereas the EN intervention only improved language scores.

With respect to indirect effects, each intervention had a significant positive indirect effect through OMCI on all four development domains at 24 months with somewhat larger indirect effects on cognitive development (Table 4). Although indirect effects through OMCI on LAZ were not statistically significant at the 5% level, confidence intervals were trending towards significance (i.e., confidence intervals were just below zero). In contrast, DDS mediated intervention effects on LAZ and cognitive, language and socio‐emotional development at 24 months. With respect to motor development, indirect effects through DDS were only significant for the RS intervention and trending towards significance for the EN and RS + EN intervention. Indirect effects on child development were larger through OMCI than DDS, whereas indirect effects on LAZ were larger through DDS than OMCI. Overall, OMCI mediated a larger proportion of total effects than DDS for all outcomes.

**Table 4:** Standardized indirect intervention effects on child growth and development through child diet and mother–child interactions

		Bias‐corrected bootstrapped 95% CI	
Pathways	*β*	Lower limit	Upper limit
*RS intervention*			
RS → DDS (12 months) → LAZ (24 months)	0.004	0.001	0.010
RS → DDS (12 months) → Cognitive development (24 months)	0.005	0.001	0.016
RS → DDS (12 months) → Language development (24 months)	0.006	0.001	0.016
RS → DDS (12 months) → Motor development (24 months)	0.002	0.000	0.008
RS → DDS (12 months) → Socio‐emotional development (24 months)	0.004	0.000	0.012
RS → OMCI (12 months) → LAZ (24 months)	0.008	−0.009	0.023
RS → OMCI (12 months) → Cognitive development (24 months)	0.056	0.031	0.091
RS → OMCI (12 months) → Language development (24 months)	0.051	0.026	0.081
RS → OMCI (12 months) → Motor development (24 months)	0.050	0.026	0.079
RS → OMCI (12 months) → Socio‐emotional development (24 months)	0.050	0.020	0.085
*EN intervention*			
EN → DDS (12 months) → LAZ (24 months)	0.008	0.003	0.016
EN → DDS (12 months) → Cognitive development (24 months)	0.010	0.002	0.023
EN → DDS (12 months) → Language development (24 months)	0.012	0.005	0.024
EN → DDS (12 months) → Motor development (24 months)	0.004	−0.001	0.012
EN → DDS (12 months) → Socio‐emotional development (24 months)	0.007	0.000	0.019
EN → OMCI (12 months) → LAZ (24 months)	0.005	−0.006	0.018
EN → OMCI (12 months) → Cognitive development (24 months)	0.039	0.021	0.063
EN → OMCI (12 months) → Language development (24 months)	0.035	0.020	0.056
EN → OMCI (12 months) → Motor development (24 months)	0.034	0.019	0.054
EN → OMCI (12 months) → Socio‐emotional development (24 months)	0.035	0.013	0.064
*RS* + *EN intervention*			
RS + EN → DDS (12 months) → LAZ (24 months)	0.009	0.003	0.018
RS + EN → DDS (12 months) → Cognitive development (24 months)	0.011	0.003	0.025
RS + EN → DDS (12 months) → Language development (24 months)	0.014	0.005	0.026
RS + EN → DDS (12 months) → Motor development (24 months)	0.005	−0.002	0.013
RS + EN → DDS (12 months) → Socio‐emotional development (24 months)	0.008	0.000	0.020
RS + EN → OMCI (12 months) → LAZ (24 months)	0.007	−0.008	0.022
RS + EN → OMCI (12 months) → Cognitive development (24 months)	0.049	0.028	0.079
RS + EN → OMCI (12 months) → Language development (24 months)	0.045	0.025	0.069
RS + EN → OMCI (12 months) → Motor development (24 months)	0.044	0.024	0.067
RS + EN → OMCI (12 months) → Socio‐emotional development (24 months)	0.044	0.018	0.076

*Note*: The null hypothesis was *β* = 0. Models for each child development domain were fit separately. Models controlled for the following enrolment characteristics: household wealth, household food security, maternal education, maternal mental health, child age, child sex, child length‐for‐age *z*‐score, and number of siblings. Models accounted for clustering and missing values.

Abbreviations: DDS, dietary diversity score; EN, enhanced nutrition; LAZ, length‐for‐age *z*‐score; OMCI, observation of mother–child interaction; RS, responsive stimulation.

These positive indirect effects together with the negative direct intervention effects at 24 months indicated the presence of competitive mediation. Specifically, the negative effect of the RS interventions on LAZ [*β* −0.015 (95% CI: −0.105, −0.008)] was cancelled out by the RS intervention's positive impacts on OMCI [*β*: 0.008 (95% CI: −0.009, 0.023)] and DDS [*β*: 0.004 (95% CI: 0.001, 0.010)]. Likewise, the negative effect of the RS + EN interventions on LAZ [*β*: −0.081 (95% CI: −0.129, −0.034)] was cancelled out by its positive effect on OMCI [*β*: 0.007 (95% CI: −0.008, 0.022)] and DDS [*β*: 0.009 (95% CI: 0.003, 0.018)].

## 4 DISCUSSION

We showed that a responsive stimulation (RS) and enhanced nutrition (EN) intervention improved child cognitive, language and motor development among young Pakistani children through child diet and mother–child interactions. Although only the EN intervention alone (as compared with the standard of care) improved children's socio‐emotional development, we observed positive indirect effects of all three interventions through both child diet and mother–child interactions. Similarly, none of the interventions improved child growth; however, we found positive indirect effects of all three interventions through child diet. These findings suggest that child diet mediated intervention effects on child growth, but both child diet and mother–child interactions mediated intervention effects on child development, and are in line with evidence indicating that the determinants of linear growth and development are only partially shared (Prado, Larson, et al., 2019). Overall, our findings confirmed that all paths included in our conceptual model were important, albeit not all estimates reached statistical significance.

A major contribution of the current study is showing that the RS and EN interventions appeared to have positive cross‐over effects on caregiver behaviours, that is, they worked through secondary mechanisms. Specifically, the EN intervention improved child development through mother–child interactions and the RS intervention improved child growth through child diet. These findings lend support to the translation hypothesis put forth by scholars that mothers are able to translate responsiveness and supportive mother–child interactions across facets (Landry et al., 2006; Nahar et al., 2009). The RS intervention improved mother–child interactions, which may have led to better feeding techniques or more responsive feeding (Nahar et al., 2009). Similarly, the EN intervention promoted responsive feeding which may have led to the improvements in mother–child interactions we observed. However, we did not measure responsive feeding or other responsiveness aspects and were therefore unable to formally test this translation hypothesis. Moreover, the mother–child interactions tool we used only captured responsive and supportive interactions in a picture book‐reading context, which is different from a feeding context that typically has clear objectives and indicators of success (e.g., child eating). Our findings should be interpreted with caution and replicated in similar samples in other LMICs. Future studies should be specifically designed to assess if, and how, caregivers apply responsive strategies across play, book‐reading, feeding and other contexts, and to start building a more substantial evidence base for or against this translation hypothesis.

Another important contribution of our study is that we were able to assess why the RS and EN interventions may have had no overall effect (as compared with the standard of care) on socio‐emotional development and child growth. With respect to child growth, we showed that the gains achieved through child diet were offset by residual negative effects of the RS and RS + EN interventions. Similar patterns emerged with respect to socio‐emotional development, though many of the direct, indirect, and total effects did not reach statistical significance. These residual negative intervention effects could be due to environmental risks not accounted for in our model. For example, if the RS intervention increased free exploration and play activities on the ground, this may have increased children's exposure to pathogens and environmental risks. Increased or persistent immune stimulation may contribute to poor child growth and socio‐emotional development (Ngure et al., 2014). Alternatively, the RS and EN inputs may have been insufficient to address existing environmental risks for poor child growth and development in this vulnerable context. With respect to child growth, intrauterine growth restriction, prematurity, and low birthweight are risk factors for growth faltering. In this study, we lacked data on birth weight and gestational age, and were unable to assess the association of linear growth independent of characteristics at birth. Overall, although consistent with prior literature showing the lack of RS effects on child growth and socio‐emotional development (World Health Organization, 2020), our findings also indicate that the mechanisms through which RS interventions affect these outcomes are still poorly understood. Future studies should collect data on all hypothesized mechanisms to help fully unpack the effects of RS interventions. Caution is also needed in designing future interventions to help minimize potential negative effects. Although baby water, sanitation and hygiene (WASH) has been proposed as one approach to address environmental risks (Ngure et al., 2014), no evidence to date exists on the feasibility, acceptability and effectiveness of integrating RS and baby WASH inputs. Evidence on successful integration of EN and household‐level WASH inputs is emerging, but effects on child growth and development are mixed (Pickering et al., 2019; Stewart et al., 2018; Tofail et al., 2018).

Several limitations of our study should be noted. First, child diet was based on maternal report, which could be subject to social desirability bias, and was limited to a single recall over the past 24 h, which did not capture food quantity or nutrient content. Moreover, although we controlled for household food security, household wealth, and maternal education, we lacked data on other community‐, household‐ and maternal‐level factors that influence child diet, for example, food availability, seasonality, and affordability, maternal awareness of children's nutritional needs, child eating skills and acceptance. Second, the mother–child interactions tool we used does not eliminate observer bias and the observed mother–child interactions may be different from regular interactions. Importantly, the tool measured mother–child interactions only in a picture book‐reading context. More work is needed to confirm its ability to measure mother–child interactions in other situations or settings. Third, omitted variables may bias our results. While we controlled for several important confounders, unmeasured and unobserved variables may confound the relations of interest, particularly those between the mediators and outcomes.

Despite these limitations, our findings have two main implications for multi‐input interventions aiming to improve child growth and development in LMICs: (1) such interventions should combine RS and EN inputs, and (2) such interventions should leverage both child diet and mother–child interactions as mechanisms. First, although we observed similar indirect effects through mother–child interactions among children exposed to the RS and RS + EN interventions, we observed larger indirect effects through child diet among children exposed to the RS + EN interventions and fewer residual negative effects. These findings confirm that combining RS and EN interventions leads to no loss of effect on child growth and development (S. M. Grantham‐McGregor et al., 2014), and that multi‐input interventions can be more effective (World Health Organization, 2020), though we did not explicitly test for additive or synergistic effects. Second, although child diet alone mediated intervention effects on child growth, both child diet and mother–child interactions mediated intervention effects on child development. Given the growing evidence that the determinants of child growth and development are only partially shared (Prado, Larson, et al., 2019), future interventions aiming to improve both outcomes should be designed to leverage both shared (e.g., child diet) and separate (e.g., mother–child interactions) mechanisms. Currently, many multi‐input interventions promote responsive stimulation and teach optimal child feeding practices. Our findings further build the case for these types of interventions by strengthening the evidence base and demonstrating that improvements in mother–child interactions and child diet translate into tangible benefits for child growth and development. The emerging evidence on cross‐over effects suggests that these two mechanisms are mutually reinforcing. Thus, interventions that promote both mechanisms will likely be more effective at improving child outcomes than interventions promoting only one of the mechanisms.

Our findings build on prior PEDS work by demonstrating that child diet and mother–child interactions mediated intervention effects on child growth and development in the first 2 years of life. Prior PEDS studies showed that maternal mental health and parental stimulation were other mechanisms through which the intervention worked (Brown et al., 2017; Jeong et al., 2019; Obradović et al., 2016). Taking all these findings together, the PEDS interventions helped build an enabling and nurturing environment which directly benefited mothers and their children both during the intervention implementation period and after the intervention ended. These findings suggest that similar types of multi‐input integrated RS and EN interventions can serve as a platform to enable, empower, and support caregivers, which in turn can improve not only child growth and development, but also child wellbeing more generally and the overall conditions in which children grow up. Future interventions in LMICs should focus on holistic approaches, which aim to improve multiple aspects of nurturing care rather than on individual child outcomes.

## CONFLICT OF INTERESTS

The authors declare that there are no conflict of interests.

## AUTHOR CONTRIBUTIONS

LB and AKY conceptualized the present analyses. AKY designed the PEDS evaluation, and AKY and SS led data collection activities. LB and DCM led the data analyses. LB drafted the manuscript. All authors contributed to interpreting and discussing the results and revising the manuscript. All authors read and approved the final version of the paper. LB had final responsibility for submitting this article for publication.

## Supporting information

Supporting information.

## Data Availability

The data that support the findings of this study are available from the corresponding author upon reasonable request.
