# Supplementary material for: Child diet and mother–child interactions mediate intervention effects on child growth and development
Source: Matern Child Nutr. 2021 Dec 14;18(2):e13308. doi: 10.1111/mcn.13308 (PMC8932723; doi:10.1111/mcn.13308)
Supplement: Supplementary file 1 — Supporting information. [file MCN-18-e13308-s001.docx]

**Supplemental Table 1** Enrolment characteristics of children included in the analytic sample and those excluded from the analytic sample

|  | Included in the analytic sample (N=1,350) | Excluded from the analytic sample (N=139) |  |
| --- | --- | --- | --- |
| Variables | % or mean ± SD | % or mean ± SD | p-value for difference between groups |
| Child age (in months) | 0.68±0.65 | 0.74±0.68 | 0.34 |
| Child is a boy | 54% | 54% | 0.93 |
| Mother has no formal education | 68% | 64% | 0.28 |
| Household wealth index | 0.01±1.00 | -0.03±0.97 | 0.67 |
| Household is food secure | 68% | 64% | 0.71 |

**Supplemental Table 2** Descriptive statistics of the 19 items comprising the Observation of Mother-Child Interaction (OMCI) tool

|  |  | N (%) | | | |
| --- | --- | --- | --- | --- | --- |
|  | Mean ± SD | 0-1 occurrences | 2-3 occurrences | 3-4 occurrences | ≥5 occurrences |
| 1. Shows positive affect for the child, for example, smiles at child, laughs with child and speaks in a soft tone to the child | 1.82±0.87 | 77 (5.75%) | 419 (31.27%) | 509 (37.99%) | 335 (25%) |
| 2. Shows negative affect for the child, for example, shows frustration with child, frowns at child and disengaged from child for more than 10 seconds | 0.04±0.2 | 1296 (96.72%) | 40 (2.99%) | 4 (0.3%) | 0 (0%) |
| 3. Shows positive touch, for example, strokes the child gently or touches the child with affection or kisses the child | 0.45±0.72 | 904 (67.46%) | 274 (20.45%) | 151 (11.27%) | 11 (0.82%) |
| 4. Shows negative touch, for example, pushes child away, roughly handles child | 0.03±0.17 | 1308 (97.61%) | 30 (2.24%) | 2 (0.15%) | 0 (0%) |
| 5. Expresses positive verbal statements, for example, praises the child for something related to picture, praises the child for something else and expresses love or affection verbally | 0.34±0.7 | 1033 (77.09%) | 179 (13.36%) | 104 (7.76%) | 24 (1.79%) |
| 6. Expresses negative verbal statements, for example, scolds child, aggressive or abusive language | 0.02±0.15 | 1315 (98.13%) | 23 (1.72%) | 2 (0.15%) | 0 (0%) |
| 7. Is sensitive of the child's needs, for example, follows child's lead, accepts child’s disinterest in book and does not force infant to play with it any longer | 1.69±0.71 | 33 (2.46%) | 515 (38.43%) | 627 (46.79%) | 165 (12.31%) |
| 8. Expands on child's prior talk, for example, adds or builds on what the child is saying and interprets what the child is saying | 0.49±0.76 | 884 (65.97%) | 287 (21.42%) | 142 (10.6%) | 27 (2.01%) |
| 9. Points and names object in book | 1.79±0.94 | 144 (10.75%) | 322 (24.03%) | 541 (40.37%) | 333 (24.85%) |
| 10. Questions child, for example, asks child to name an object in the picture and asks child to point to an object | 1.69±0.94 | 151 (11.27%) | 411 (30.67%) | 485 (36.19%) | 293 (21.87%) |
| 11. Answers child's question or request | 0.5±0.89 | 948 (70.75%) | 209 (15.6%) | 94 (7.01%) | 89 (6.64%) |
| 12. Helps child to maintain interest, for example, tries to create interest in the pictures through verbalizing, helps the child explore the book and actively comments on the infant’s actions | 1.51±0.8 | 130 (9.7%) | 530 (39.55%) | 547 (40.82%) | 133 (9.93%) |
| 13. Smiles, laughs with caregiver | 1.24±0.99 | 373 (27.84%) | 437 (32.61%) | 365 (27.24%) | 165 (12.31%) |
| 14. Shows excitement and enjoyment like clapping | 1.09±1.1 | 564 (42.09%) | 295 (22.01%) | 284 (21.19%) | 197 (14.7%) |
| 15. Crying, frowning, frustrated | 0.32±0.72 | 1066 (79.55%) | 163 (12.16%) | 66 (4.93%) | 45 (3.36%) |
| 16. Explores the book for a significant time (for at least 1 minute), for example, looks at pictures, tries to turn page, points to picture and verbalizes to picture | 1.76±0.84 | 77 (5.75%) | 438 (32.69%) | 556 (41.49%) | 269 (20.07%) |
| 17. Continues in spite of distractions | 1.45±0.97 | 243 (18.13%) | 468 (34.93%) | 410 (30.6%) | 219 (16.34%) |
| 18. Vocalizing or producing words | 1.22±1.09 | 453 (33.81%) | 367 (27.39%) | 293 (21.87%) | 227 (16.94%) |
| 19. Expressing enjoyment while exploring together | 1.16±0.90 | 366 (27.31%) | 491 (36.64%) | 390 (29.1%) | 93 (6.94%) |

**Supplemental Table 3** Biserial correlations of the variables included in the model

| Variables | 1 | 2 | 3 | 4 | 5 | 6 | 7 | 8 | 9 | 10 | 11 | 12 | 13 | 14 | 15 | 16 | 17 | 18 | 19 | 20 |
| --- | --- | --- | --- | --- | --- | --- | --- | --- | --- | --- | --- | --- | --- | --- | --- | --- | --- | --- | --- | --- |
| LAZ, 12 mo | − |  |  |  |  |  |  |  |  |  |  |  |  |  |  |  |  |  |  |  |
| LAZ, 24 mo | 0.79** | − |  |  |  |  |  |  |  |  |  |  |  |  |  |  |  |  |  |  |
| Cog, 12 mo | 0.29** | 0.28** | − |  |  |  |  |  |  |  |  |  |  |  |  |  |  |  |  |  |
| Cog, 24 mo | 0.28** | 0.32** | 0.36** | − |  |  |  |  |  |  |  |  |  |  |  |  |  |  |  |  |
| Lang, 12 mo | 0.25** | 0.22** | 0.48** | 0.30** | − |  |  |  |  |  |  |  |  |  |  |  |  |  |  |  |
| Lang, 24 mo | 0.31** | 0.33** | 0.36** | 0.67** | 0.35** | − |  |  |  |  |  |  |  |  |  |  |  |  |  |  |
| Motor, 12 mo | 0.33** | 0.32** | 0.55** | 0.35** | 0.43** | 0.36** | − |  |  |  |  |  |  |  |  |  |  |  |  |  |
| Motor, 24 mo | 0.36** | 0.37** | 0.40** | 0.71** | 0.32** | 0.75** | 0.40** | − |  |  |  |  |  |  |  |  |  |  |  |  |
| Soc-Emo, 12 mo | 0.20** | 0.19** | 0.36** | 0.21** | 0.37** | 0.23** | 0.36** | 0.23** | − |  |  |  |  |  |  |  |  |  |  |  |
| Soc-Emo, 24 mo | 0.21** | 0.23** | 0.19** | 0.35** | 0.19** | 0.34** | 0.21** | 0.37** | 0.19** | − |  |  |  |  |  |  |  |  |  |  |
| DDS, 12 mo | 0.15** | 0.18* | 0.16** | 0.18*** | 0.15*** | 0.21*** | 0.19*** | 0.16*** | 0.11*** | 0.11*** | − |  |  |  |  |  |  |  |  |  |
| OMCI, 12 mo | 0.16** | 0.14** | 0.38** | 0.32** | 0.43** | 0.34** | 0.34** | 0.31** | 0.39** | 0.18** | 0.13** | − |  |  |  |  |  |  |  |  |
| LAZ, 0 mo | 0.49** | 0.43** | 0.15** | 0.13** | 0.06* | 0.14** | 0.22** | 0.18** | 0.07** | 0.14** | 0.02 | 0.05 | − |  |  |  |  |  |  |  |
| Child is a boy | -0.06* | -0.01 | 0.05 | 0.04 | -0.03 | 0.00 | 0.04 | 0.05 | 0.00 | 0.02 | -0.01 | 0.01 | -0.04 | − |  |  |  |  |  |  |
| Child age | 0.05 | 0.06* | -0.05 | 0.04 | -0.09** | 0.02 | -0.02 | 0.04 | -0.19** | 0.02 | 0.05 | -0.07* | 0.04 | 0.00 | − |  |  |  |  |  |
| Household wealth | 0.25** | 0.28** | 0.17** | 0.24** | 0.18** | 0.22** | 0.18** | 0.26** | 0.11** | 0.10** | 0.08** | 0.12** | 0.10** | -0.01 | 0.12** | − |  |  |  |  |
| Maternal education | -0.2** | -0.22** | -0.13** | -0.18** | -0.13** | -0.18** | -0.10** | -0.17** | -0.07** | -0.14** | -0.07* | -0.13** | -0.04 | 0.00 | -0.11** | -0.34** | − |  |  |  |
| Maternal SRQ-20 | -0.04 | -0.06* | -0.10** | -0.09** | -0.07* | -0.10** | -0.08** | -0.11** | -0.08** | -0.04 | -0.06* | -0.03 | -0.05 | -0.01 | 0.05 | -0.10** | 0.09** | − |  |  |
| Household size | -0.11** | -0.11** | -0.09** | -0.12** | -0.09** | -0.11** | -0.04 | -0.11** | -0.08** | -0.08** | 0.00 | -0.05 | 0.07* | 0.01 | 0.00 | -0.13** | 0.16** | 0.14** | − |  |
| Household food secure | 0.13** | 0.17** | 0.15** | 0.18** | 0.17** | 0.20** | 0.13** | 0.19** | 0.13** | 0.15** | 0.08** | 0.13** | 0.01 | -0.03 | -0.05 | 0.25** | -0.23** | -0.20** | -0.12** | − |

*Note.* LAZ = length-for-age Z-score; BISD = Bayley Scales of Infant and Toddler Development; Lang = BSID Language Composite Score; Cog = BSID Cognitive Composite Score; Motor = BSID Motor Composite Score; Soc-Emo = BSID Socio-emotional Composite Score; DDS = dietary diversity score; OMCI = observation of mother-child interaction; SRQ = self-reported questionnaire.

* *p* < .05. ** *p* < .01.

**Supplemental Table 4** Standardized direct effects of main paths in the conceptual framework

|  |  | Bias-corrected bootstrapped 95% CI | |
| --- | --- | --- | --- |
| Paths | *β* | Lower limit | Lower limit |
| Direct intervention effects on mediators at 12 months |  |  |  |
| RS → DDS | 0.077 | 0.012 | 0.145 |
| RS → OMCI | 0.435 | 0.349 | 0.510 |
| EN → DDS | 0.147 | 0.077 | 0.225 |
| EN → OMCI | 0.300 | 0.225 | 0.374 |
| RS+EN → DDS | 0.170 | 0.099 | 0.247 |
| RS+EN → OMCI | 0.383 | 0.301 | 0.456 |
| Direct effects of mediators on outcomes at 24 months |  |  |  |
| DDS → LAZ | 0.051 | 0.018 | 0.087 |
| DDS → Cognitive development | 0.065 | 0.011 | 0.123 |
| DDS → Language development | 0.080 | 0.033 | 0.128 |
| DDS → Motor development | 0.028 | -0.013 | 0.070 |
| DDS → Socio-emotional development | 0.047 | -0.004 | 0.102 |
| OMCI → LAZ | 0.018 | -0.021 | 0.056 |
| OMCI → Cognitive development | 0.129 | 0.072 | 0.187 |
| OMCI → Language development | 0.117 | 0.063 | 0.173 |
| OMCI → Motor development | 0.115 | 0.061 | 0.168 |
| OMCI → Socio-emotional development | 0.114 | 0.041 | 0.187 |

*Note.* The null hypothesis was *β*=0. Models for each child development domain were fit separately. Models controlled for the following enrolment characteristics: household wealth, household food security, maternal education, maternal mental health, child age, child sex, child length-for-age z-score, and number of siblings. Models accounted for clustering and missing values. RS = responsive stimulation; MAD = minimum acceptable diet; OMCI = observation of mother-child interaction; EN = enhanced nutrition; LAZ = length-for-age Z-score.

**Supplemental Table 5** Standardized direct intervention effects on child outcomes at 24 months

|  |  | Bias-corrected bootstrapped 95% CI | |
| --- | --- | --- | --- |
| Pathways | *β* | Lower limit | Lower limit |
| Direct RS effects |  |  |  |
| RS → LAZ | -0.051 | -0.105 | -0.008 |
| RS → Cognitive development | 0.226 | 0.153 | 0.306 |
| RS → Language development | 0.232 | 0.153 | 0.324 |
| RS → Motor development | 0.181 | 0.111 | 0.260 |
| RS → Socio-emotional development | -0.069 | -0.146 | 0.006 |
| Direct EN effects |  |  |  |
| EN → LAZ | -0.009 | -0.061 | 0.042 |
| EN → Cognitive development | 0.035 | -0.045 | 0.110 |
| EN → Language development | 0.134 | 0.052 | 0.221 |
| EN → Motor development | 0.050 | -0.024 | 0.121 |
| EN → Socio-emotional development | 0.013 | -0.062 | 0.093 |
| Direct RS+EN effects |  |  |  |
| RS+EN → LAZ | -0.081 | -0.129 | -0.034 |
| RS+EN → Cognitive development | 0.112 | 0.040 | 0.179 |
| RS+EN → Language development | 0.172 | 0.087 | 0.264 |
| RS+EN → Motor development | 0.104 | 0.029 | 0.180 |
| RS+EN → Socio-emotional development | -0.050 | -0.129 | 0.024 |

*Note.* The null hypothesis was *β*=0. Models for each child development domain were fit separately. Models controlled for the following enrolment characteristics: household wealth, household food security, maternal education, maternal mental health, child age, child sex, child length-for-age z-score, and number of siblings. Models accounted for clustering and missing values. RS = responsive stimulation; LAZ = length-for-age Z-score; EN = enhanced nutrition.
